# A double barrier memristive device

**DOI:** 10.1038/srep13753

**Published:** 2015-09-08

**Authors:** M. Hansen, M. Ziegler, L. Kolberg, R. Soni, S. Dirkmann, T. Mussenbrock, H. Kohlstedt

**Affiliations:** 1 Nanoelektronik, Technische Fakultät Kiel, Christian-Albrechts-Universität Kiel, Kiel 24143, Germany; 2 Ruhr University Bochum, Faculty of Electrical Engineering and Information Technology, Institute of Theoretical Electrical Engineering, Bochum D-44780, Germany

## Abstract

We present a quantum mechanical memristive Nb/Al/Al_2_O_3_/Nb_x_O_y_/Au device which consists of an ultra-thin memristive layer (Nb_x_O_y_) sandwiched between an Al_2_O_3_ tunnel barrier and a Schottky-like contact. A highly uniform current distribution for the LRS (low resistance state) and HRS (high resistance state) for areas ranging between 70 μm^2^ and 2300 μm^2^ were obtained, which indicates a non-filamentary based resistive switching mechanism. In a detailed experimental and theoretical analysis we show evidence that resistive switching originates from oxygen diffusion and modifications of the local electronic interface states within the Nb_x_O_y_ layer, which influences the interface properties of the Au (Schottky) contact and of the Al_2_O_3_ tunneling barrier, respectively. The presented device might offer several benefits like an intrinsic current compliance, improved retention and no need for an electric forming procedure, which is especially attractive for possible applications in highly dense random access memories or neuromorphic mixed signal circuits.

Memristive devices have emerged as promising candidates in the field of non-volatile data storage for future information technology where the device resistance depends on the history of the applied voltage^1^,^2^,^3^,^4^. Due to their simple two terminal capacitor-like layer sequence (metal-insulator-metal), highly scalable crossbar arrays and multilevel memory structures have been proposed where memristive devices might overcome technical and physical scaling limits of modern semiconductor devices^5^,^6^,^7^. Their binary and analog properties qualify them as promising building blocks for *in-situ*-computing^8^. Apart from memory and logic applications, the use of memristive devices as artificial synapses in neuromorphic circuits is intensively discussed, focusing on bio-inspired artificial neuronal networks^9^,^10^. In general, today’s research on memristive devices and networks is characterized by numerous elegant system concepts for novel memories, programmable logic units and neuromorphic circuits limited only by a lack of reliable devices and a thorough understanding of the involved switching mechanisms. Nevertheless, the steady progress in memristive device performance in recent years could close the gap between promising computing concepts and the hardware realizations in the near future.

Although the underlying physical mechanism is often unclear, the majority of memristive devices involve the random creation of one or more conductive filaments, resulting in a poor switching reproducibility and a high device-to-device variability^6^,^11^,^12^,^13^. Moreover, most memristive devices require an initial and individual electrical forming step, additionally complicating their use in crossbar architectures and complex mixed-signal circuits.

Interface-based devices may overcome these restrictions, because uniform interface effects lead to a homogeneous change in resistance, avoiding the randomness generated by electroforming or filament growth^14^,^15^,^16^,^17^,^18^,^19^,^20^. Most of the investigated interfacial devices are oxide-metal junctions, where the resistive switching mechanism results from changes at a Schottky-like contact^15^,^21^. A less common approach uses junctions consisting of a tunnel barrier and a memristive layer, where the change in resistance results from a varying electron tunneling probability^17^,^18^,^22^,^23^. To explain the not completely understood resistance change in interface-based devices, two rather different models are usually considered: The first model is related to the concept of charge injection, where traps within the memristive layer or at the metal interface are charged and discharged, resulting in a high- and low-resistances state, respectively^14^,^24^,^25^,^26^,^27^,^28^. In the second model, the applied electric field is sufficient to move ions within the memristive layer, leading to a change in interfacial properties and consequently changing the overall device resistance. Besides interface effects, contributions from the memristive layer itself (e.g. local chemical bounds, oxide phases, doping, local heating effects and so on) may affect or oppose the resistive switching, making a thorough analysis of the underlying mechanism more complicated. Therefore, scaling down the thickness of the memristive layer to the length scale of a single electron wave may provide an opportunity to avoid the stated contributions of the memristive layer, while the use of a second barrier might restrict switching effects to interfacial contributions and to derive a physical model of the resistance switching mechanisms.

Here, a double barrier device with an ultra-thin memristive layer sandwiched between a tunnel barrier and a Schottky-like contact is presented. The layer sequence of the device is Al/Al_2_O_3_/Nb_x_O_y_/Au, with a thickness of 1.3 nm for the Al_2_O_3_ tunnel barrier and 2.5 nm for the Nb_x_O_y_ layer. In order to get a deeper understanding of the particular interfacial contributions to the observed switching characteristics, single barrier devices were fabricated, i.e. an Al/Al_2_O_3_/Nb_x_O_y_/Nb tunnel junction excluding the Schottky contact and an Nb/Nb_x_O_y_/Au Schottky contact without the tunneling barrier. Based on the experimental results an equivalent circuit model was developed, which shows evidence that the Nb_x_O_y_ layer may act as an ionic/electronic (mixed) conductor, where the switching mechanism is related to mobile ions within the Nb_x_O_y_. This might offer several benefits. For example, the properties of the Al_2_O_3_ tunnel barrier could define the lower resistance boundary (i.e. the LRS) of the junction. In particular, amorphous Al_2_O_3_ is known to be a “good” tunnel barrier (i.e., elastic electron tunnelling dominates the transport) where the barrier thickness can be effectively controlled during growth^29^. The tunnel barrier thickness acts as a current limiter and represents an essential design parameter as will be explained in detail. The tunnel barrier and the gold electrode define chemical barriers for the ionic species, confining them within the Nb_x_O_y_. A saturation of the ion density (number of ions per area) at either interface will define the LRS and HRS. No current compliance is needed, due to the self-limited ion assembly at either interface. The finite activation energies of the ionic species will lead to a frozen (memory) resistance state in case of zero bias and will therefore improve the data retention compared to a purely electronic switching mechanism, which face a voltage-time dilemma^30^.

## Results

### Device structure

**Figure 1:**
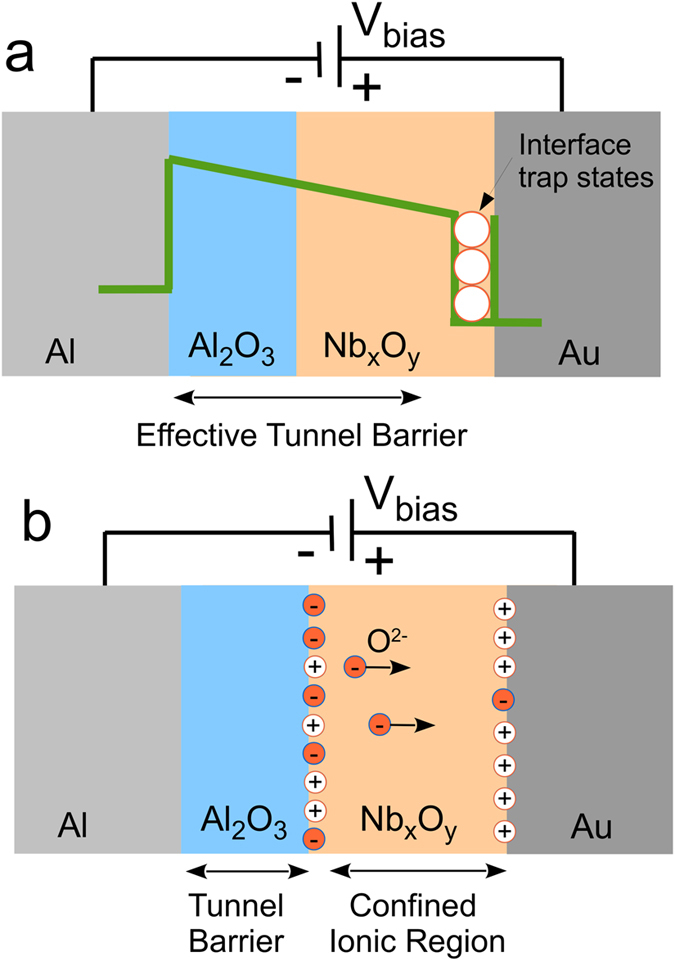
Two models to describe the memristive double barrier tunnel junctions. (**a**) Simplified cross-sectional view of the memristive tunnel junctions. Here, trap states within the Nb_x_O_y_ are assumed. The filling and emptying of traps by injected electrons varies the amount of charge in the Nb_x_O_y_ layer and therefore the resistance. (**b**) An alternative model to (**a**). Under forward bias voltages V_bias_ oxygen ions (orange circles) can move inside the Nb_x_O_y_ layer, where their diffusion region is confined by the Al_2_O_3_ layer and the Nb_x_O_y_/Au interface. Both, the model in (**a**) as well as the model in (**b**) describe the memristive I–V characteristics.

Figure 1 shows the cross-section of the double barrier Al/Al_2_O_3_/Nb_x_O_y_/Au memristive device. The thickness of the Al_2_O_3_ tunnel barrier is 1.3 nm and that of the Nb_x_O_y_ layer 2.5 nm. In general, two rather different physical mechanisms may describe the memristive characteristics of this double barrier device. In Fig. 1(a), Nb_x_O_y_ acts as a trapping layer for electrons, where localized electronic states within the Nb_x_O_y_ layer are filled or emptied depending on the applied bias voltage polarity. Therefore, the amount of charge within this layer depends on the history of the applied bias voltage, where charged traps and discharged traps will represent the high- and low-resistances state, respectively. The first charge trapping model, originally used to describe resistive switching in metal-insulator-metal (MIM) Al/SiO (20 nm–300 nm)/Au junctions, was developed from Simmons and Verderber^31^.

In contrast to the charge injection model of Fig. 1(a), Nb_x_O_y_ acts as an ionic/electronic (mixed) conductor in the model shown in Fig. 1(b). Here, Nb_x_O_y_ represents a solid state electrolyte, while Al_2_O_3_ serves as a tunnel barrier. By applying a bias voltage, oxygen ions (within the Nb_x_O_y_) drift towards the tunnel barrier or Au interface in dependence on their charge and mobility. The redistribution of the ionic species will affect essential interfacial parameters (e. g. density of states, local barrier height, barrier thickness and so on) at the Al_2_O_3_/Nb_x_O_y_ and the Nb_x_O_y_/Au (Schottky) interface simultaneously. By applying an opposite bias, the original ion distribution should be obtained. As a consequence, the electronic transport, i.e. the device resistance, will be altered in accordance to the local ion distribution leading to memristive *I–V* characteristics. We would like to emphasize that the charge injection and the mobile ion model (Fig. 1(a,b)) will be discussed below with respect to the experimental findings.

### Resistive switching behaviour

**Figure 2:**
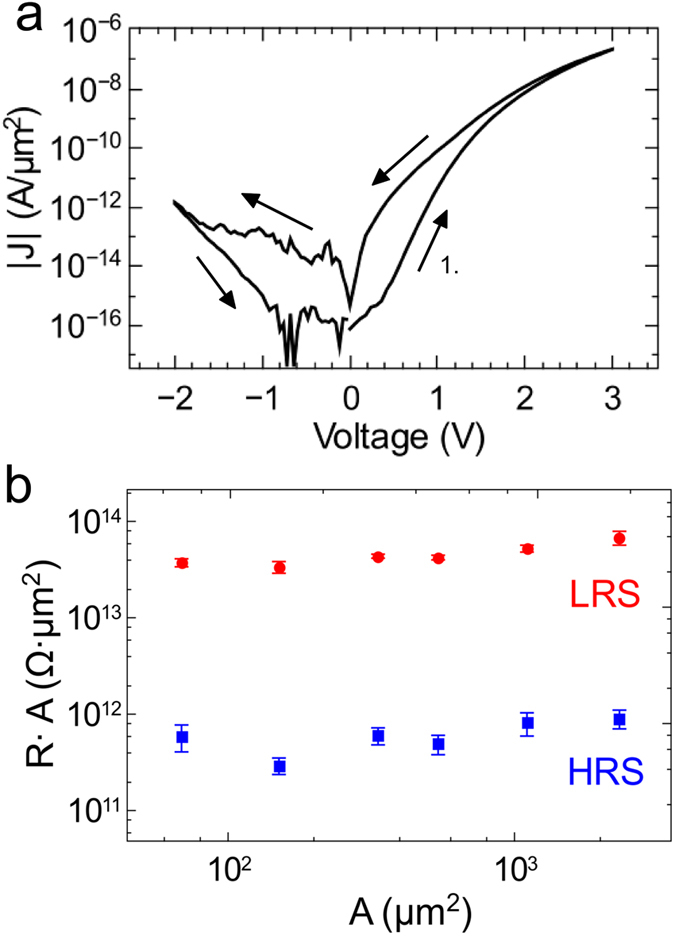
Resistive switching characteristics of the memristive double barrier device. (**a**) Absolute current density *|J|* as function of the applied bias voltage. (**b**) The area-resistance product vs. junction-area curve of the double barrier device measured at 0.5 V indicates a homogeneous area dependent charge transport. The error bars are obtained from 5 cells of each area. Junction areas were confirmed with optical microscopy.

A representative current-voltage (*I*–*V*) characteristic of the double barrier memristive device is depicted in Fig. 2(a). Neither an initial forming procedure nor a current compliance was used. Instead, a linear voltage sweep was applied to the Au electrode, while the current was measured simultaneously. In particular, the voltage was ramped linearly from 0 V to 2.8 V in order to set the device from the high resistance state (HRS) to the low resistance state (LRS), as marked by arrows in Fig. 2(a). To set the device resistance back to the initial HRS the voltage was ramped linearly from 2.8 V to −2 V and afterwards increased to 0 V. As a result, a pinched hysteresis loop of a bipolar memristive device was obtained. The fluctuations for small currents under negative bias indicate the current resolution of our set-up rather than physically relevant mechanisms. The most apparent feature of the memristive hysteresis is the asymmetry between positive and negative bias, which can be attributed to the Schottky-like Nb_x_O_y_/Au contact. Moreover, an important feature of our double barrier memristive device is the gradual resistance change rather than abrupt resistance jumps. An abrupt jump in the device resistance during voltage sweeps may indicate a filamentary-driven resistance switching effect, while gradual changes may result from homogeneously changed interface properties^14^,^16^. This suggestion is supported by the *R × A* vs. *A* plot shown in Fig. 2(b). For junctions with areas ranging from 70 μm^2^ to 2300 μm^2^, *R × A* for the high and low resistance states is independent of the device area, which suggests a homogeneous switching mechanism.

### Interface barrier contributions

For interface-based memristive behavior, the charge transport through the tunneling barrier has to be dominated by elastic tunneling rather than trap induced tunneling or interfacial trap states within the Al_2_O_3_ barrier. This requires a nearly defect free, highly stable, and electrically high-quality tunnel barrier. Additionally, it requires that the memristive behavior originates from changes in the Nb_x_O_y_ layer, while the Al_2_O_3_ layer is stable under a changed contact resistance at the Nb_x_O_y_ interface. In particular, Al_2_O_3_ ranks among the best tunnel barriers for this purpose. In the field of superconductivity, Al_2_O_3_ is intensively used as a tunnel barrier in Josephson junctions, where the Nb/Al/Al_2_O_3_ technology is the prevailing technology^29^. Moreover, the sputtered Nb_x_O_y_ has been found to be amorphous by using X-ray diffraction measurements. Therefore, the Al_2_O_3_ can be assumed to be of higher quality than the Nb_x_O_y_.

**Figure 3:**
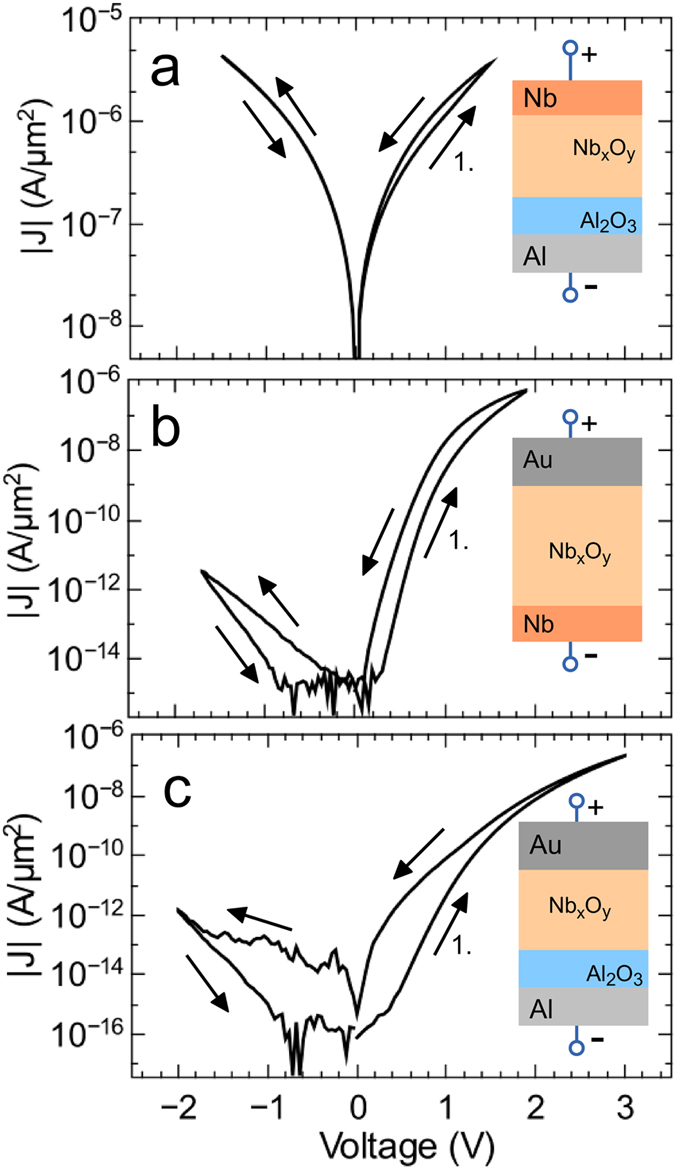
Interface contributions. Absolute current density *|J|* versus applied bias voltage of (**a**) an Al/Al_2_O_3_/Nb_x_O_y_ tunnel junction, (**b**) an Nb/Nb_x_O_y_/Au Schottky contact and (**c**) for comparison the Al/Al_2_O_3_/Nb_x_O_y_/Au double barrier device. Insets: Simplified cross-sectional view of the devices.

To get a deeper understanding of the transport mechanism, two additional devices were investigated to separate the particular interfacial contributions. Therefore, Al/Al_2_O_3_/NbO_x_ tunnel junctions excluding the Schottky contact, as well as Nb/Nb_x_O_y_/Au Schottky contacts without the tunnel barrier were prepared, as shown in Fig. 3. Here, Nb is used as the electrode to keep the difference in work function between the electrode and Nb_x_O_y_ layer low. The obtained *I*–*V* curves are compared in Fig. 3(a,b). While memristive behavior is clearly visible for the Nb/Nb_x_O_y_/Au contact, no change in the device resistance behavior was observed for Al/Al_2_O_3_/Nb_x_O_y_ tunnel junctions (Fig. 3(a)). This indicates that the Nb_x_O_y_/Au Schottky-like interface contributes to the resistive switching observed in the double-barrier device. Memristive devices with oxide-metal Schottky contacts have been studied extensively, and the origin of the resistive switching is supposed to be the modulation of the Schottky barrier height^15^. Nonetheless, the aforementioned observations indicate that the two interfaces Al_2_O_3_/Nb_x_O_y_ and Nb_x_O_y_/Au cannot be treated as separate entities and involve a very strong mutual interdependence. However, the following analysis considers the influence of both interfaces individually, while taking into account that both mechanisms should be treated simultaneously.

**Figure 4:**
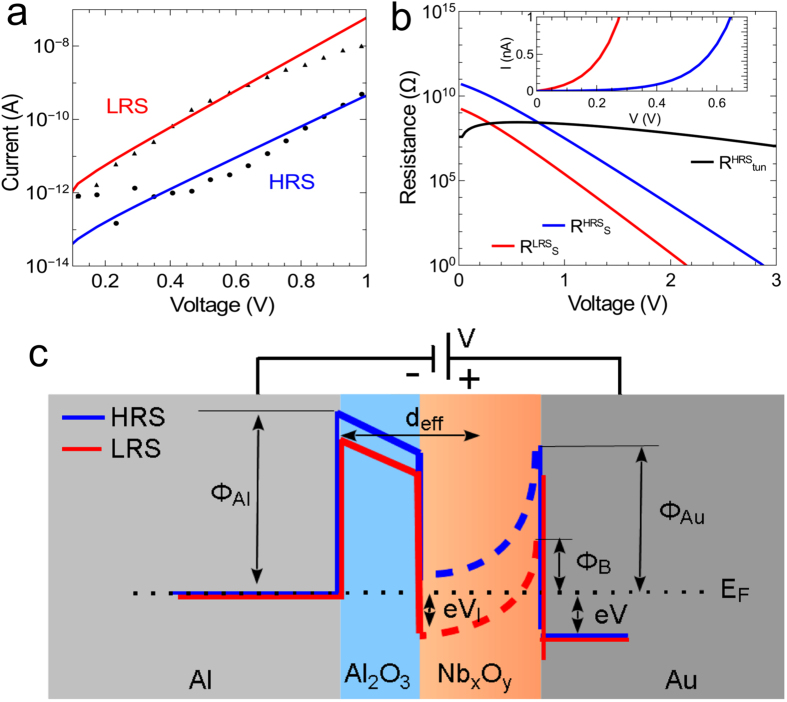
Schematic of the electronic band structure variations. (**a**) *I–V* characteristics of the LRS and HRS branch of the double barrier device in the reverse voltage regime of the Schottky contact (device area is normalized to 1 μm^2^). Solid lines are data fits according to Equation 1 to extract the Schottky barrier height *ϕ*_*B*_. (**b**) Comparison between the Schottky contact resistance at the LRS and HRS branch and the tunnelling resistance. Inset: Shift of the diode forward current onset. (**c**) Schematic electronic band diagram of the double barrier structure for the LRS (red line) and HRS (blue line). During the transition from the HRS to LRS, moving oxygen ions cause an decrease of the interfacial potential V_I_ by a down shift of the interfacial band in Nb_x_O_y_ (dashed line). Further, the effective barrier tunnel width *d*_*eff*_ and the apparent barrier heights *ϕ*_*Al*_, and *ϕ*_*Au*_ of ,respectively, the Al electrode, and Au contact are decreased.

In order to study the influence of the Schottky interface, the thermionic emission theory was employed to get information from *I*–*V* data (cf. Fig. 4(b)). In this theory, a Schottky contact is described by a set of analytical expressions, where the Schottky diode current for forward bias voltages is defined as^32^,^33^


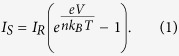


Where *k*_*B*_ and *T* are the Boltzmann constant and temperature, respectively, while *n* is the ideality factor which describes the derivation from the ideal current. The reverse current *I*_*R*_is given by


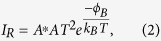


where *ϕ*_*B*_ is the Schottky barrier height, *A* the junction area, and A^*^ the effective Richardson constant, which is *1.20173 10*^*6*^ *Am*^*−2*^*K*^*−2*^. The reverse current is dominated by the lowering of the Schottky barrier. If, however, the apparent barrier height *ϕ*_*B*_ at the Schottky interface is reasonably smaller than the conductive band gap of the insulator, the reverse current decreases gradually with the applied negative bias and it follows from Equations 1, 2 that^32^


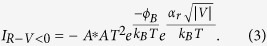


Here, α_r_ denotes a device dependent parameter which is used to describe the experimentally observed reverse voltage dependence. By using *ϕ*_*B*_ and *n* as fit parameters, Equation 1 is fitted in the low forward bias voltage regime (*V* < 1 V) to the LRS and HRS branches of the measured *I*–*V* curve. The resulting fit curves are shown in Fig. 4(a). The apparent Schottky barrier height decreases from 0.62 eV to 0.54 eV and the ideality factor from 4.1 to 3.5 when the device resistance is decreased. In particular, the decrease of the ideality factor with increasing forward current suggests surface effects at the Nb_x_O_y_/Au interface, which could be the reason for the observed lowering of the apparent Schottky barrier height^20^,^19^ in agreement with recent findings^15^.

Besides the Schottky interface, contributions arising from the tunnelling barrier have to be taken into account to better understand the charge transport in the device. The internal electrical field distribution is of particular interest, since this determines the effective interfacial potentials within the Nb_x_O_y_ layer. Figure 4(b) compares the contact resistance of the Schottky interface for the HRS (blue line) and LRS (red line) to the tunneling resistance of the Al_2_O_3_ layer. For the calculation of the tunnelling resistance, we used the tunneling current formula from Simmons^34^:


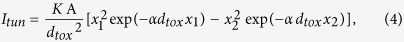


where 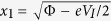, 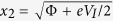, *K* = *6.32 10*^*10*^ *V/s*, Φ is the apparent barrier height of the tunnelling oxide (Φ = *(ϕ*_*Al*_ + *ϕ*_*NbO*_*)/2* = *3.1* *eV*), *A* the normalized device area (*A* = 1 μ*m^2^),* and 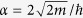 (*m*: free electron mass; *ћ* Planck’s constant divided by 2π). *V*_*I*_ is the resulting voltage across the Al_2_O_3_ tunnel barrier and d_*tox*_ is the thickness of the Al_2_O_3_ layer (d_*tox*_ = *1.3 *nm). As a result, most of the applied voltage drops across the Schottky barrier for bias voltages below 0.5 V (LRS) and 1.0 V (HRS), while the tunnelling resistance of the Al_2_O_3_ layer is getting more important for voltages above 0.5 V (LRS) and 1.0 V (HRS). The inset in Fig. 4(b) shows the state dependent diode forward voltages, V_HRS_ = *0.65 V* and *V*_*LRS*_ = *0.255 V,* at a forward current of *1 nA*. Thus, the electron transport at low voltages is limited by the Schottky barrier and at higher voltages by the tunnelling barrier. Figure 4(b) shows a comparison for a constant tunnelling distance. However, due to the small thickness of the Al_2_O_3_ and the Nb_x_O_y_ layer, the properties of both interfaces, Al_2_O_3_/Nb_x_O_y_ and Nb_x_O_y_/Au cannot be considered independent. Any change in the electronic distribution within the Nb_x_O_y_ layer will affect both interfaces simultaneously. Thus, the device can be only described by taking the properties of both interfaces into account, since both interfaces are interwoven via the Nb_x_O_y_ layer. Our experimental data indicates a homogeneous area-dependent charge transport mechanism. This might be especially important in the case where mobile oxygen-ions are involved in the resistance switching process, as described in the model in Fig. 1(b). A variation of the oxygen concentration in the Nb_x_O_y_ solid state electrolyte layer affects interfacial properties of both the Al_2_O_3_/Nb_x_O_y_ as well as the Nb_x_O_y_/Au boundary. The macroscopically measured memristive I–V curve of a junction is a result of a delicate superposition of electronic and ionic effects at both interfaces. One obvious possibility is the change of the effective tunnelling thickness in accordance to the oxygen concentration at the Al_2_O_3_/Nb_x_O_y_ contact:


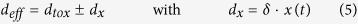


Here, δ denotes the maximum variation of the effective distance and *x(t)* is the state variable of the memristive process, which ranges between 0 and 1. Figure 4(c) shows a schematic electronic band diagram for the double barrier device, which assumes an effective tunneling distance and a varying Schottky barrier height. In particular, the HRS (blue line) and LRS (red line) band profiles are shown, which differ due to the changed contact resistance at the Nb_x_O_y_/Au interface. This leads to a variation of the band structure, which itself influences the tunnelling process.

### Device model

Although the fabricated devices and electrical characterizations fit the scenario described above, the experimental results can be explained by both models, i.e. charging and discharging of interfacial trap states and mobile oxygen ions. Moreover, by considering the local electric field and possible distribution of ions with respect to the electron tunneling transport, the electrical properties of the device and their interdependencies are not obvious at all and require a closer look from a theoretical point of view. Therefore, equivalent circuit models for the devices are often developed, which provides a more thorough understanding of the device functionality^35^,^36^,^37^,^38^,^39^,^40^,^41^.

**Figure 5:**
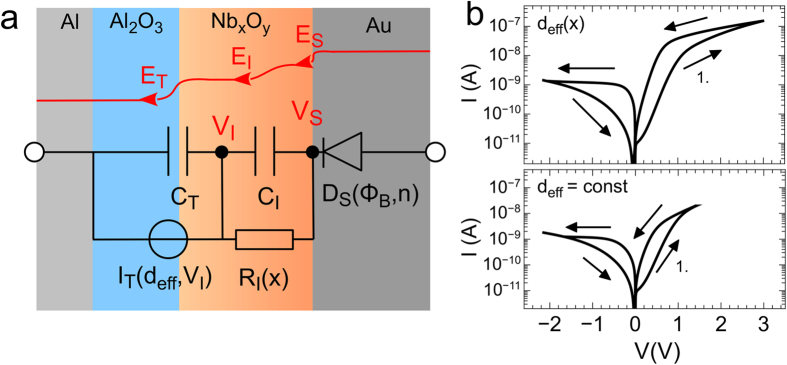
Device model. (**a**) Equivalent circuit model of a double barrier memristive device. The tunnelling current is defined by a current source supplying the current according to Equation 4. The Schottky barrier is taken into account as a diode according to Equation 1. The capacitance of the tunnelling barrier is C_T_, while C_I_ represents the capacitance of the Nb_x_O_y_ layer. The oxygen-ion migration, which changes the interfacial potential V_I_, is expressed in the model as a variable resistance R_I_(x) parallel to the Nb_x_O_y_ layer capacitance C_I_. The local electrical field strength E_S_, E_I_, and E_T_ due to the applied voltage are indicated as a red line. (**b**) Calculated I–V curve for a variable tunneling distance and (**c**) for a constant tunneling distance.

Figure 5(a) shows the equivalent circuit which was used to model the scenario from Fig. 1(a), where both, mobile oxygen ions and interfacial trap states, are responsible for the observed resistive switching mechanism. The calculated I–V curve shown in Fig. 5(b) contains the main experimental recorded characteristics, such as an asymmetric pinched hysteresis, a high resistance at small voltages and the current saturation at higher voltages. In contrast, Fig. 5(c) shows the simulated I–V curve for a constant effective tunneling distance *d*_*eff*_. In agreement with our experimental findings (cf. Fig. 3(b,c)) this decoupling of the particular interfacial interactions reduces the width of the memristive hysteresis, indicating that mobile oxygen-ions are involved in the resistance switching process and that the two interfaces cannot be treat separately.

For the device model, the capacitances of the Nb_x_O_y_/Au interface *C*_*I*_ and the tunnelling barrier *C*_*T*_ are modelled in a capacitive divider. The values of *C*_*I*_ and *C*_*T*_ have been estimated from capacitance measurements. Therefore, devices with an Nb_x_O_y_ layer thickness ranging from 1 nm to 20 nm were fabricated and the total capacitance was measured. By using a linear regression and extrapolating to 0 nm Nb_x_O_y_, the values *C*_*T*_ = *2.07* × *10*^*−14*^ *F*/*μm^2^* and *C*_*I*_ = *1.74 *× *10*^*−14*^ *F/μm^2^* were found. The electron tunnelling is implemented in the model by a voltage driven current source, which depends on the effective tunnelling distance *d*_*eff*_ (see Equation 5) and the interfacial potential V_*I*_. The potential change of the Nb_x_O_y_ resistance is taken into account by the resistance *R*_*I*_(x), which depends on the memristive state variable *x(t)*. Additionally, the Schottky contact resistance is accounted for by using a Schottky diode *D*_*S*_ described by Equations 1, 2, 3. This diode defines the potential *V*_*S*_(*ϕ*_*B*_, *n*), which is a function of the memristive state dependent Schottky barrier height (*ϕ*^*HRS*^_*B*_ = *0.62* *eV* and *ϕ*^*LRS*^_*B*_ = *0.54* *eV*) and the ideality factors (*n*^*HRS*^ = *4.1* and *n*^*LRS*^ = *3.5*). At negative bias voltages, the Schottky contact induced potential *V*_*S*_(*ϕ*_*B*_, *n*) is strongly influenced by the reverse current (cf. Equation 3), so that we assume a state variable dependent reset of *ϕ*_*B*_ according to *ϕ*_*B*_ = *x/x*_*max*_*ϕ*^*LRS*^_*B*_ + (1 − *x/x*_*max*_*) ϕ*^*HRS*^_*B*_, where *x*_*max*_ is the maximal value of *x* during the set process and x varied between 0 and 1. For the set process we are using *ϕ*^*HRS*^_*B*_ and *ϕ*^*LRS*^_*B*_ estimated from Fig. 4(a).

The interfacial potential V_*I*_ is used as the reference potential of the equivalent circuit, so that the total capacitance of the device is *C*_*tot*_ = *C*_*T*_ + *C*_*I*_ (since they are in parallel according to the reference potential) and V_*I*_ thus reads


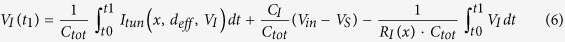


where *V*_*in*_ is the external applied bias voltage. Hence, the specific value of the interfacial potential *V*_*I*_ modulates the tunnelling current and is responsible for the electron injection and the local field strength. The profile of the electrical field is sketched in Fig. 5(a) (red curve). Here, *E*_*T*_, *E*_*I*_, and E_S_ are, respectively, the local electrical field strengths at the tunnelling barrier, across the Nb_x_O_y_ layer, and at the Schottky contact at the Au interface. While *E*_*T*_ depends on the effective tunnelling distance according to *E*_*T*_ = *V*_*I*_*/d*_*eff*_, we assume that the Nb_x_O_y_ layer thickness *d*_*NbO*_ defines the region *E*_*I*_ = *(V*_*I*_ − *V*_*S*_*)/d*_*NbO*_. In addition, *E*_*I*_ depends on the Schottky potential *V*_*S*_. For our device model we assume that mobile oxygen ions are involved in the resistance switching mechanisms by calculating the memristive state variable x(t) using the simple one-dimensional voltage driven memristor model of Ref. 4,


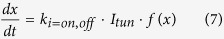


where *k*_*on*_ = 35 × 10^2^ (*As*)^*−1*^ and *k*_*off*_ = 37 × 10^4^ (*As*)^−1^ are constants for positive and negative bias voltages, while *f(x)* is the window function defined in Ref. 42. Therefore, the resistance change within the Nb_x_O_y_ layer can be calculated by 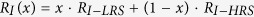, where *R*_*I-HRS*_ = 7 *MΩ* and *R*_*I-LRS*_ = *6* *MΩ* are the interface resistances for the HRS and LRS, respectively and *x* changes between 0 and 1.

## Discussion

The most important feature of our device is the double barrier separated by the ultra-thin solid state electrolyte Nb_x_O_y_, which restricts the resistive switching to interface effects. The use of homogenous, interface effects as the origin of memristive switching avoids the formation of active current paths (conductive filaments) through the device. This avoids the drawbacks of initial electroforming steps and allows the targeted development of memristive devices by a controlled modification of interfacial potentials. Therefore, the interplay between electron tunneling, oxygen ion diffusion inside the Nb_x_O_y_ layer, and interfacial state variations at the Schottky contact must be balanced by the local electrical fields.

**Figure 6:**
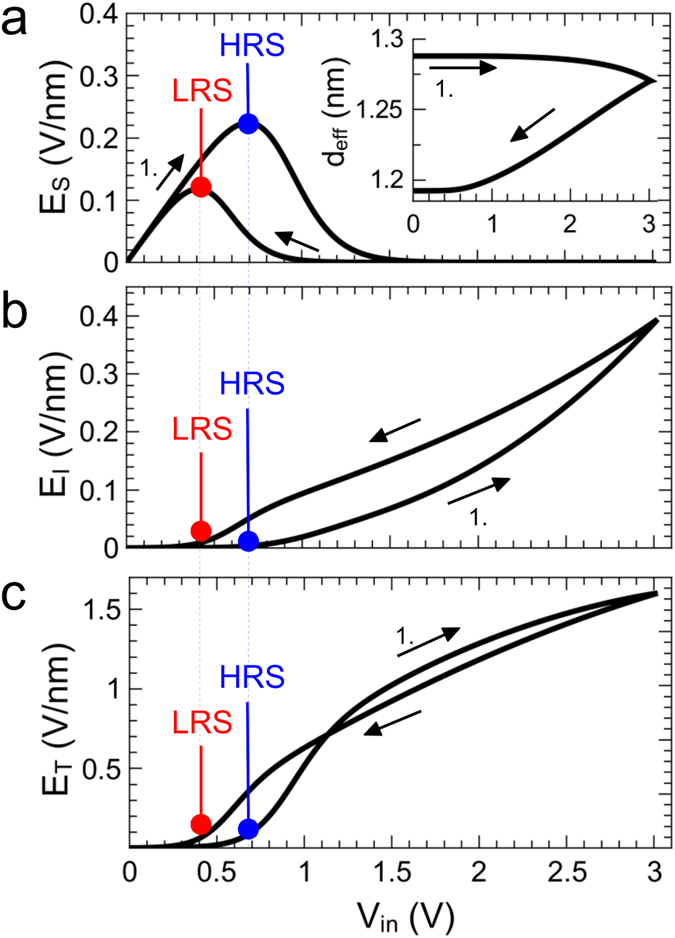
Local electrical field strengths. Calculated electrical fields across the Schottky contact *E*_*S*_, the *Nb*_*y*_*O*_*x*_ layer *E*_*I*_ (**b**), and across the tunnelling barrier *E*_*T*_ (**b**) during a positive voltage sweep. The onset current of the Schottky diode for the LRS and HRS are marked in red and blue, respectively. The black arrows show the direction of the voltage sweep. Inset: Corresponding change in effective tunneling distance.

Figure 6 shows the calculated electric field strengths for positive bias voltages across the tunneling barrier E_T_, the interfacial layer of the double barrier device E_I_, and the Schottky contact E_S_. At low bias voltages the electrical field across the interfacial layer (Fig. 6(b)) and the tunneling layer (Fig. 6(c)) is nearly zero, since the current is blocked by the Schottky contact (Fig. 6(a)). In other words, the Schottky diode defines a threshold voltage for our device, which has to be exceeded to change the resistance of the device. This can be seen from the inset of Fig. 6(a), which shows the change of the effective tunneling distance as a function of the applied external voltage. Here, the resulting electrical field across the Nb_x_O_y_ layer is too small to affect either ion diffusion or electron injections in the reverse diode regime. It is worth to mention that the device threshold additionally depends on the particular resistance state of the device, as marked by blue and red dotted lines in Fig. 6(a–c) for the HRS and LRS, respectively. This opens the possibility to adjust the device threshold by the memristive state of the device, which may be of interest in neuromorphic circuits to emulate threshold dependent plasticity processes, or for ultra dense packet crossbar memory arrays to suppress sneak-path leakage currents^43^.

If the applied bias voltage is increased in the forward regime of the Schottky diode D_S_, the electric field *E*_*T*_ increases exponentially. While *E*_*T*_ saturates at higher voltages, *E*_*I*_ shows no saturation effects. Moreover, *E*_*I*_ exhibits memristive behavior in a way that the field strength with increasing voltage is always smaller than on the way back, while *E*_*T*_ decreases and crosses itself at *1.1* V, when the external voltage is ramped back to zero (cf. Fig. 5(c)). Regarding the device model shown in Fig. 5(a), we can explain the observed saturation from the interplay between the effective tunneling distance and the interfacial potential *V*_*I*_. In particular, the increase of the tunneling current stems from the decrease of the tunneling resistance due to the reduced effective tunneling distance (cf. inset Fig. 5(a)). Since the interfacial potential *V*_*I*_ is increasing according to Equation 6 with a rising tunnelling current *I*_*tun*_ and a decreasing Schottky contact voltage *V*_*S*_, the field strength *E*_*I*_ is increasing too, according to *E*_*I*_ = *(V*_*I*_ − *V*_*S*_)/*d*_*NbO*_. However, since *E*_*T*_ is decreasing with an increasing *V*_*I*_, the tunneling interface acts as an intrinsic current compliance. Thus, no external current compliance has to be set, as it is typically the case for filamentary based memristive devices to protect the device from a dielectric breakdown. It is worth to mention that thin insulating layers as tunneling barriers have been used already to incorporate memristive cells into high density cross-type array structures^44^. Due to their non-linear I–V characteristic, tunneling barriers act as access devices, where an intrinsic current compliance improves the device endurance by suppressing a too high device current during voltage application.

**Figure 7:**
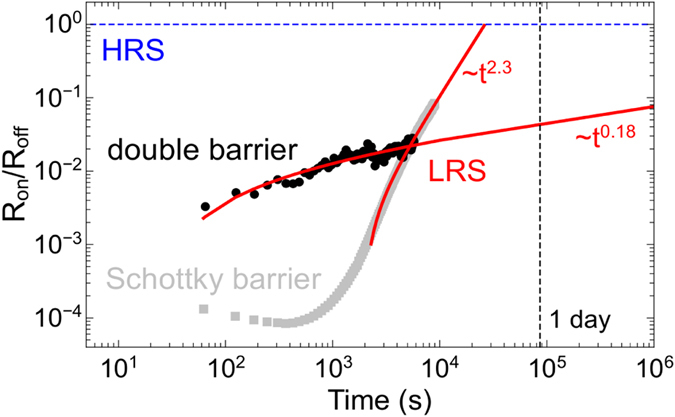
Retention characteristics. Time dependence of the LRS (data points) compared to the HRS (blue dashed line) for the Nb_x_O_y_/Au Schottky contact of Fig. 2(a) (gray data points) and the double barrier device (black data points). For the readout of the resistance state read pulses of 0.5 V every 60 s were applied. Red lines are data fits used to extrapolate retention times. Inset: retention characteristic for the double barrier device within the first 500s.

However, by referring to the two initially described microscopic models of trap charging and discharging (Fig. 1(a)) versus mobile ions (or oxygen vacancies, Fig. 1(b)), the inner field strength *E*_*I*_ is in the order of 10^−1^ V/nm and consequently allows filling and emptying of traps as well as the drift of oxygen-ions within the Nb_x_O_y_ layer as realistic scenarios. While the developed equivalent circuit models shows evidence that oxygen-ion diffusion are involved in the switching mechanisms, the impact of interfacial trap states cannot be ruled out. A closer look at the retention characteristic might be helpful to gain further insight. In Fig. 7 the retention characteristic of the memristive double barrier device is compared to the retention characteristic of the single Schottky barrier memristive device presented in Fig. 3(b). For the retention time measurements the devices were first set to their LRS by ramping the bias voltage to *2.9* *V* and *2.0* V for the memristive double barrier device and memristive Schottky contact, respectively. Afterwards the resistance was recorded by applying voltage pulses of *0.5* *V* for *2* *s* every *60* *s* and measuring the current. The double barrier device shows an increase in resistance in the first *500* *s* (black data points in Fig. 7), while afterwards the resistance increase is significantly less pronounced and shows an *R*_on_/*R*_off_ ratio of more than one order of magnitude after one day. In contrast to the retention characteristic of the double barrier memristive device, the Schottky barrier device exhibited virtually no increase in the device resistance up to 700 s (gray data points in Fig. 7) but increased drastically afterwards. Hence, the introduction of the Al_2_O_3_ barrier led to a significantly improved retention characteristic.

These obviously different retention behaviours can be quantitatively determined by fitting the experimental data to a *t*^*β*^ power law (red lines in Fig. 7), a procedure typically used to characterise high *k*-dielectrics^44^. While the resistance ratio R_on_/R_off_ of the Schottky barrier device follows a power law of the form t^3.56^, the double barrier device follows for the first 600 s a t^0.65^ power law and then changes to t^0.18^. Although we cannot give a final explanation for the long retention time in case of the double barrier device, possibly both initially referred effects (cf. Fig. 1) are essential to explain the observed retention characteristics. However, from our investigation, the following picture seems to be the most likely: Right after switching the device resistance to the low resistance state, previously emptied traps are filled by electronic charge carriers, leading to an increase in resistance. After approximately 100 s this mechanism fades out and is followed by a second, independent, ionic driven process. During switching, oxygen vacancies are pushed towards the Au interface and lower the contact resistance. The further increase towards the high resistance state can be explained by a partial back-diffusion of oxygen vacancies, leading nevertheless to a considerably longer retention time in comparison to the Schottky barrier device. Nonetheless, further investigations are necessary to clarify the relevance of electronic and ionic effects in ultrathin double barrier devices.

For possible applications of the presented device, we see interesting possibilities in the field of neuromorphic computing^9^,^10^, where high resistances are desired in order to reduce the overall power consumption of the whole system. Moreover, the extremely high resistance at low voltages (<1V) make our device in particular interesting for crossbar-architectures, since it requires no selector device^43^, as already mentioned above. However, we would like to mention that the scalability of the device is restricted by the relative high device resistances, which results from the Au (Schottky) contact. Here, the use of other electrode materials might lead to lower resistances without affecting the presented double barrier device mechanism.

In conclusion, a double barrier memristive device was realized with a highly uniform current distribution for the high and low resistance states, which indicates a non-filamentary based resistive switching mechanism. We have shown evidence that the use of an ultra-thin Nb_x_O_y_ solid state electrolyte layer of 2.5 nm sandwiched between an Au (Schottky) contact and an Al_2_O_3_ tunneling barrier restricts the resistive switching mechanism to interfacial effects where both barriers are involved. This may lead to the observed drastically improved retention characteristic compared to the single barrier Schottky contact devices and may be based on confined oxygen ion diffusion within the sandwiched Nb_x_O_y_ layer. An equivalent circuit model of the device was developed, which confirms our experimental findings and shows evidence that mobile oxygen-ions are involved in the resistance switching process. In order to come to a quantitative description of the resistance switching process, further investigation is necessary to study the influence of the second claimed scenario of charged traps within the Nb_x_O_y_ layer. However, we showed that the controlled modification of interfacial potentials allows the development of homogenous resistive switching mechanisms, which is essential for a wider application of memristive devices in future digital and complex analog memory circuits.

## Methods

### Sample preparation

Memristive tunneling junctions were fabricated on 4-inch Si wafers with 400 nm of SiO_2_ (thermally oxidized) using a standard optical lithography process. The junctions are arranged in 1 mm × 1 mm cells across the wafer, containing 6 different contact sizes ranging from 70 μm^2^ to 2300 μm^2^. The devices were fabricated using the following procedure: First of all, the multilayer (including top- and bottom-electrode) is deposited without breaking the vacuum using DC magnetron sputtering. The Al_2_O_3_ tunnel barrier was fabricated by depositing Al which was afterwards partially oxidized *in-situ*, the Nb_x_O_y_ layer was deposited by reactive sputtering in an O_2_/Ar-atmosphere. Following the subsequent lift-off, the junction area was defined by etching the top electrode using wet etching (potassium iodide, for Au) and dry etching (reactive ion etching with SF_6_, for Nb). The etched parts were then covered with thermally evaporated SiO to insulate the bottom electrode from the subsequently deposited Nb-wiring to contact the top electrode.

### *I–V* measurements

All measurements were performed using an Agilent E5260 source measurement unit. Current-voltage measurements (*I–V* curves) were obtained by sweeping the applied voltage and measuring the device current simultaneously.

### Capacitance measurements

In order to determine the layer capacitance for the Al_2_O_3_ tunneling barrier and Nb_x_O_y_ solid state electrolyte, capacitance measurements were done using a precision HP4284A LCR meter. The measurements were performed at room temperature using a bias voltage of 0.5 V and a sinusoidal signal of 0.3 V (peak-to-peak) at 50 kHz. To determine the capacities of the Al_2_O_3_ and Nb_x_O_y_-layers, 5 samples with Nb_x_O_y_ thicknesses ranging from 1 to 20 nm were fabricated. For each of these samples, the capacity was measured for all contact areas (70 μm^2^ to 2300 μm^2^). From each contact size, about 20 devices were measured. The spread in capacity for each area was typically in the order of few percents (which already includes the device-specific variance). To calculate the total capacitance for each sample, the slope of the C vs. area plot was taken, which always showed perfect area dependence and a negligible linear offset.

## Additional Information

**How to cite this article**: Hansen, M. *et al.* A double barrier memristive device. *Sci. Rep.* **5**, 13753; doi: 10.1038/srep13753 (2015).
